# Comparison of anticipated and actual control group outcomes in randomised trials in paediatric oncology provides evidence that historically controlled studies are biased in favour of the novel treatment

**DOI:** 10.1186/1745-6215-15-481

**Published:** 2014-12-10

**Authors:** Veronica Moroz, Jayne S Wilson, Pamela Kearns, Keith Wheatley

**Affiliations:** Cancer Research UK Clinical Trials Unit, School of Cancer Sciences, University of Birmingham, Vincent Drive, Edgbaston, Birmingham, B15 2TT UK; MRC Midland Hub for Trials Methodology Research, University of Birmingham, Vincent Drive, Birmingham, B15 2TT UK

**Keywords:** Bias, Historically controlled studies, Paediatric oncology, Rare diseases

## Abstract

**Background:**

Historically controlled studies are commonly undertaken in paediatric oncology, despite their potential biases. Our aim was to compare the outcome of the control group in randomised controlled trials (RCTs) in paediatric oncology with those anticipated in the sample size calculations in the protocols. Our rationale was that, had these RCTs been performed as historical control studies instead, the available outcome data used to calculate the sample size in the RCT would have been used as the historical control outcome data.

**Methods:**

A systematic search was undertaken for published paediatric oncology RCTs using the Cochrane Central Register of Controlled Trials (CENTRAL) database from its inception up to July 2013. Data on sample size assumptions and observed outcomes (timetoevent and proportions) were extracted to calculate differences between randomised and historical control outcomes, and a one-sample *t*-test was employed to assess whether the difference between anticipated and observed control groups differed from zero.

**Results:**

Forty-eight randomised questions were included. The median year of publication was 2005, and the range was from 1976 to 2010. There were 31 superiority and 11 equivalence/noninferiority randomised questions with time-to-event outcomes. The median absolute difference between observed and anticipated control outcomes was 5.0% (range: -23 to +34), and the mean difference was 3.8% (95% CI: +0.57 to +7.0; *P* = 0.022).

**Conclusions:**

Because the observed control group (that is, standard treatment arm) in RCTs performed better than anticipated, we found that historically controlled studies that used similar assumptions for the standard treatment were likely to overestimate the benefit of new treatments, potentially leading to children with cancer being given ineffective therapy that may have additional toxicity.

**Electronic supplementary material:**

The online version of this article (doi:10.1186/1745-6215-15-481) contains supplementary material, which is available to authorized users.

## Background

Randomised controlled trials (RCTs) are considered the gold standard for the evaluation of medical treatments, as the process of randomisation provides an unbiased comparator group with which to compare the novel treatment [1]. Despite this, historically controlled studies (HCSs) are commonly undertaken in paediatric oncology (PO). This is due to a widespread belief that RCTs cannot be performed in rare diseases, because there will be too few patients [2]. This belief persists despite the well-known potential biases in HCSs; that is, other factors change over time, which introduces confounding.

Trial sample size is the planned number of patients to be included in a study, and its determination is important in clinical trial design. Elements of the sample size calculation include anticipated outcomes for the primary outcome measure in the control and experimental groups, along with the likelihood of detecting or missing an effect of this size (type I error (α) and type II error (β)). These assumptions should be reported—though sometimes they are not—in the main trial publication. Our aim was to compare the outcomes of the control groups in RCTs in PO with those anticipated in the sample size calculations in the trial protocols. The rationale was that, had these RCTs been performed as HCSs instead, the available outcome data that were used to calculate the sample size in the RCTs would have been used as the HCS outcome data.

## Methods

### Search strategy

The sample of trials was derived from a systematic search for published PO RCTs using the Cochrane Central Register of Controlled Trials (CENTRAL) database from the database’s inception up to July 2013. The search terms were ‘randomi?ed’ plus the disease name in all fields. For diseases which affect both children and adults, the search was restricted to the paediatric population. Articles in a foreign language were excluded. The search results were assessed for eligibility by a single reviewer on the basis of the title and abstract, and they were checked by a second reviewer.

### Inclusion criteria

Superiority, equivalence and noninferiority RCTs that reported either time-to-event or dichotomous outcomes were included. Dichotomous outcomes were restricted to ‘tumour response’, as this is a common primary outcome in clinical trials in cancer. Both completed trials (that is, those that achieved target sample size) and trials that had been stopped early were included. In publications in which the authors reported more than one randomised question (RQ), each question was considered separately. In the case of 2×2 factorial and three-arm designs, each randomisation arm was included as a separate RQ because two separate clinical decisions would be made in these trial designs. For diseases that affect both adults and children, only trials including predominantly children were considered, though trials with a small proportion of adults were included. To calculate both the absolute and relative differences between the randomised and historical control (HC) comparisons, data on sample size assumptions and observed outcomes were required; any RQ not including these was excluded.

### Data extraction

A data extraction sheet was designed to record the following data: (1) disease search term, (2) year of publication, (3) trial type (superiority, equivalence or noninferiority), (4) control and experimental treatments, (5) whether the trial included any adults, (6) primary outcome (as per the endpoint used in sample size calculations) and type of primary outcome (dichotomous or time to event), (7) anticipated control rate of events, (8) anticipated treatment effect, (9) types I and II errors, (10) target and recruited number of patients (as per the primary outcome analysis), (11) observed control and experimental arm event rates, (12) observed hazard ratio, (13) absolute difference between arms and (14) *P*-value (and whether the results were statistically significant for the primary outcome). Where the authors presented more than one sample size calculation (for example, the trial had been redesigned for a different outcome based on a data monitoring committee recommendation), the original assumptions were taken. Where authors presented the observed event rate that was measured at time points different from those specified in the sample size calculation, we estimated the rates for the prespecified time point based on Kaplan-Meier survival plots. If there were any design ambiguities in the published articles, the original protocols were consulted where possible. Data extraction was checked by an independent second reviewer; discrepancies were resolved by discussion or taken to a third, blinded reviewer.

### Analysis

#### Absolute differences

Absolute control arm differences were calculated using the anticipated control rate from the sample size calculation and the observed control rate from the results section of the trial report (observed - anticipated control) to measure the accuracy of the assumptions. We investigated the absolute differences for the randomised and HC comparison by looking at the differences between the experimental treatment arm and both the observed control rate and the anticipated control rate reported in the sample size calculation. Sensitivity analyses were performed by excluding RQs that formed a three-arm trial and/or trials with a factorial design. A one-sample *t*-test was used to assess whether the difference between anticipated and observed control groups differed from zero. Spearman’s correlation coefficients were used to quantify the correlations between variables. Disease prognosis for survival outcomes was calculated as estimated observed treatment and control outcomes for 3 years, assuming an exponential distribution.

#### Relative differences

For dichotomous outcomes, risk ratios (RRs) were calculated. For both randomised and HC comparison, we estimated RRs by dividing the event rate of a control group (observed or anticipated) by the event rate in the experimental arm (RR < 1 represents a benefit for the experimental arm). For time-to-event outcomes, hazard ratios (HRs) were used to assess relative differences between treatment and control groups. The presented HRs for the primary analysis were used if reported. If only the number of events in each arm (or in total) were presented in the RQ publications with corresponding *P*-values for the Cox model and the Mantel-Haenszel and logrank tests, we used methods described by Tierney and colleagues [3] to estimate HRs for the randomised comparison. In cases where data were available only for proportions of survival at a particular time point, we estimated the HR using the formula ln(treatment survival)/ln(observed control survival). For the HC comparison, we estimated HRs by calculating ln(treatment survival)/ln(anticipated control survival). The analysis was undertaken using Stata version 11 software (StataCorp, College Station, TX, USA).

## Results

### Searches

**Figure 1:**
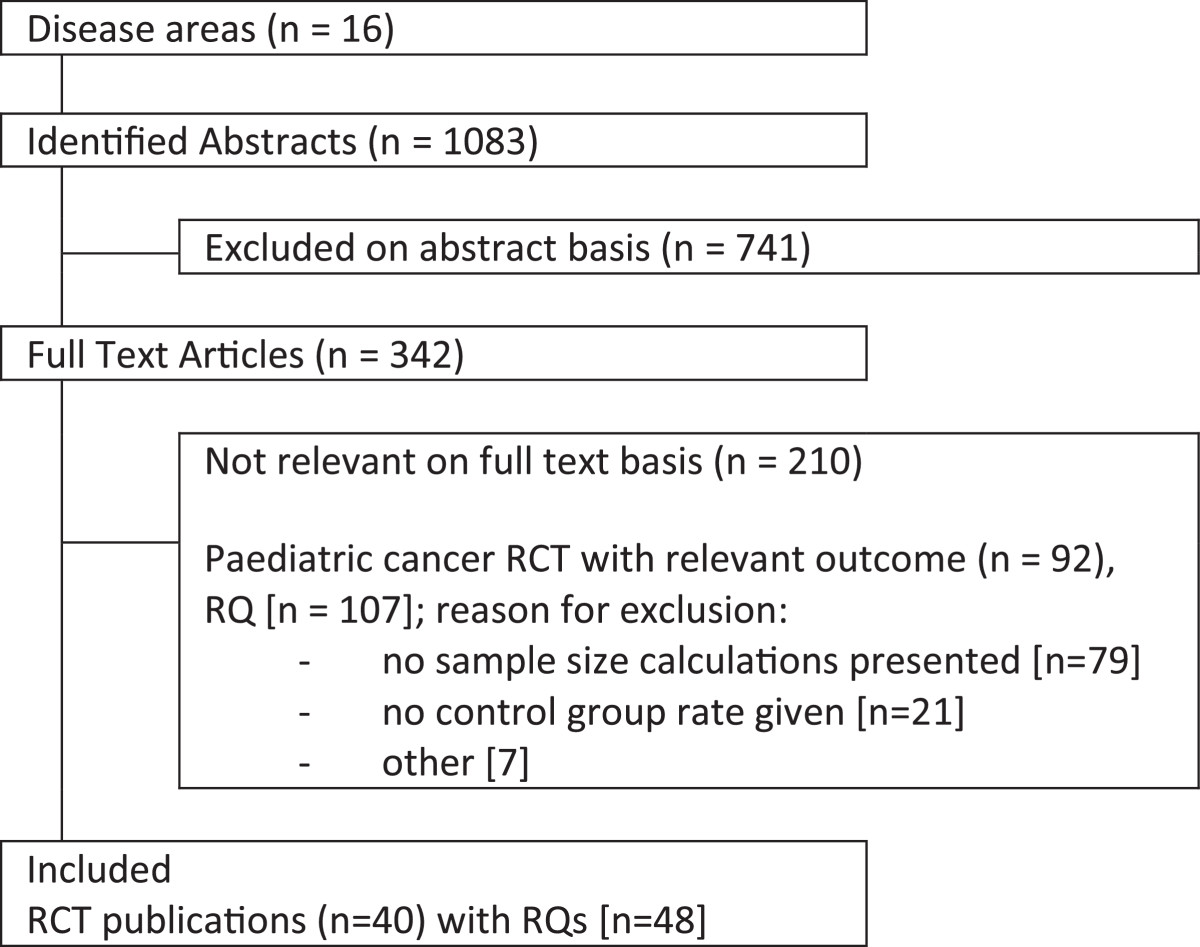
**Flowchart of the trial selection process.** RCT, Randomised controlled trial; RQ, Randomised question. Note: () represent publications and [] represent RQs.

Fifteen disease areas were searched electronically, which returned 1,083 abstracts. Forty publications with forty-eight RQs were included in the analysis (Figure 1). Lymphoma yielded the most RQs, accounting for 28% of all the RQs (Table 1).

**Table 1:** **Included randomised questions by searched term**

Search term	Randomised questions	
	***n***	(%)
Acute lymphoblastic leukaemia	6	13
Acute myeloid leukaemia	2	4
Ependymoma	0	0
Ewing’s sarcoma	2	4
Germ cell	0	0
Glioma	0	0
Hepatoblastoma	0	0
Langerhans cell histiocytosis	1	2
Lymphoma	13	27
Medulloblastoma	4	8
Neuroblastoma	4	8
Osteosarcoma	6	13
Retinoblastoma	0	0
Rhabdomyosarcoma	7	15
Wilms’ tumour	3	6
Total	48	100

### Characteristics of randomised questions

Table 2 describes the characteristics of the included RQs. The median year of publication was 2005, and the range was from 1976 to 2010 (Additional file 1). There were 31 superiority and 11 equivalence/noninferiority RQs with time-to-event outcomes. Time-to-event outcomes included event-free (a combination of progression, relapse, second malignancy, remission failure and/or death due to any cause), disease-free, relapse-free, metastasis-free, failure-free, progression-free and overall survival. The endpoint definitions used were those employed in each trial. There were six superiority trials with dichotomous primary endpoints, which included histologic, overall, rapid, complete and partial response variables. The median α and power were standard at 5% and 80%, respectively. There were six trials that had power of less than 80% and four with α above 5%. The median delta (Δ) and noninferiority margins were 15% (range: 6% to 25%) and 10% (range: 7% to 17%), respectively. The mean total design sample size was 240 (range: 92 to 1,800), and the recruited sample size was 221 (range: 36 to 2,618). Ten RQs included patients older than 22 years of age. Five RQs were parts of factorial trials [4–6], and two were parts of a three-arm trial [7].

**Table 2:** **Characteristics of the included randomised questions**

Characteristics of included RQs (***N*** = 48)	Values
Year of publication (based on 40 publications), median (range)	2005 (1976 to 2010)
Trial type and primary outcome type	
Superiority, timetoevent	31
Equivalence/noninferiority, timetoevent	11
Superiority, dichotomous	6
Power	
Median (range)	80% (73% to 99%)
Number of trials <80%	6
α	
Median (range)	5% (5% to 20%)
Number of trials >5%	3
Δ (superiority), median (range)	15% (6% to 25%)
Noninferiority margin, median (range)	10% (7% to 17%)
Total design sample size, median (range)	240 (92 to 1,800)
Total recruited sample, median (range)	221 (36 to 2,618)

Ten RQs gave ‘extra’ sample size calculations. Six of these gave calculations for a secondary outcome and/or calculations for the actual recruited number of patients [5, 8–12]; two gave power for the same sample size for a different Δ [13, 14]; and one trial was redesigned for a different primary outcome based on data monitoring committee recommendations [15]. One trial gave sample size calculations for two outcomes (overall and event-free survival (EFS)), and a blinded reviewer selected EFS for use in the analysis [16]. Two trials were stopped early, and one employed a 2:1 allocation ratio. Owing to the availability of protocols within the trials unit, the protocols for three RQs [4, 17] were checked for the time point of the anticipated control rate. The observed control rates for the prespecified time point were estimated using available Kaplan-Meier plots for 17 RQs.

For 2 of 48 RQs, the *P*-values for the primary comparison were not presented. Nine superiority and four noninferiority/equivalence RQs yielded a *P*-value <0.05 (eight superiority RQs favoured controls, two showed equivalence and one concluded noninferiority).

### Absolute difference for control group

The results described in this section are based on 47 RQs, as two of the randomised comparisons came from a three-arm trial; hence, only one control was used to compare two separate treatments.

**Figure 2:**
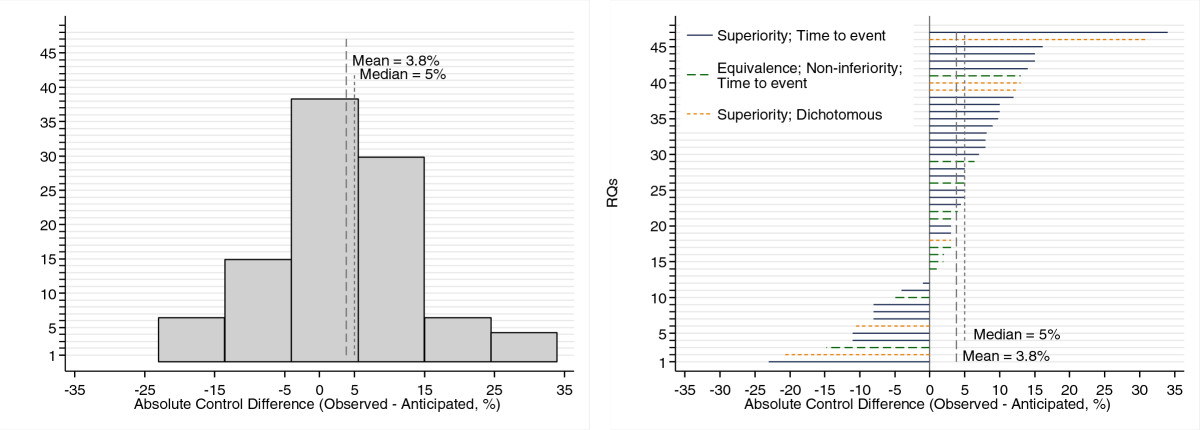
**Distribution of absolute differences between observed and anticipated control (%).** RQ, Randomised question.

Figure 2 shows the distribution of absolute differences between the observed and anticipated control group, where a positive change means that the observed control arm did better than anticipated. In 34 trials, the observed control group did better than anticipated (in 12 cases, there was ≥10% absolute difference). The observed control group did worse (by ≥10% in 6) in 12 trials, and the outcome was the same as anticipated in 1 trial. The median absolute difference between the observed control and anticipated control outcomes for all 47 trials was 5.0% (range: -23 to +34), and the mean difference was 3.8% (95% confidence interval (CI): +0.57 to +7.0); this finding was statistically significant (*P* = 0.022). A sensitivity analysis excluding five RQs that were parts of factorial trials did not change the results. For time-to-event outcomes, the median and mean differences were 5.0% (range: -23 to +34) and 4.4% (95% CI: +0.40 to +8.4), respectively, in the superiority trials; it was 3.0% (range: -15 to +13) and 1.8% (95% CI: -2.9 to +6.5), respectively, in equivalence and noninferiority trials. For trials with a dichotomous endpoint, the median and mean differences were 7.7% (range: -21 to +31) and 4.6% (95% CI: -15 to +24), respectively.

**Figure 3:**
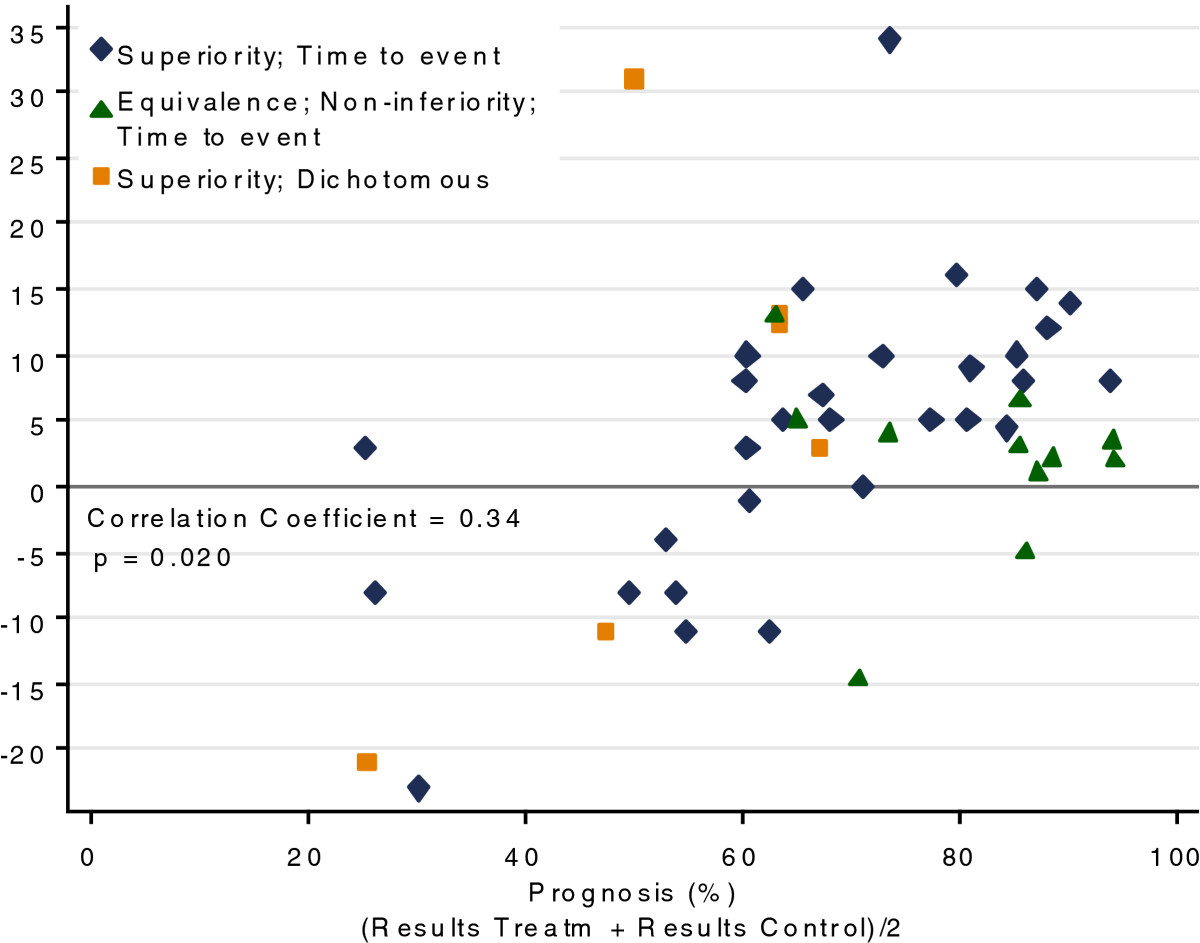
**Scatter plot for control difference and disease prognosis (100% implies a good prognosis).**

The absolute differences between the observed and the anticipated controls and trial size (number of recruited patients) were not related, with a Spearman’s correlation coefficient of 0.04 (Additional file 2). Similar results were found for the year of trial publication, with a correlation coefficient -0.07 (Additional file 2). There was a positive association between the control group differences and the prognosis of the disease (calculated as the average of observed treatment and control outcomes), with a correlation coefficient of 0.34, *P* = 0.020 (Figure 3); that is, the trials with better prognosis had larger positive differences.

### Absolute differences for control versus experimental groups

**Figure 4:**
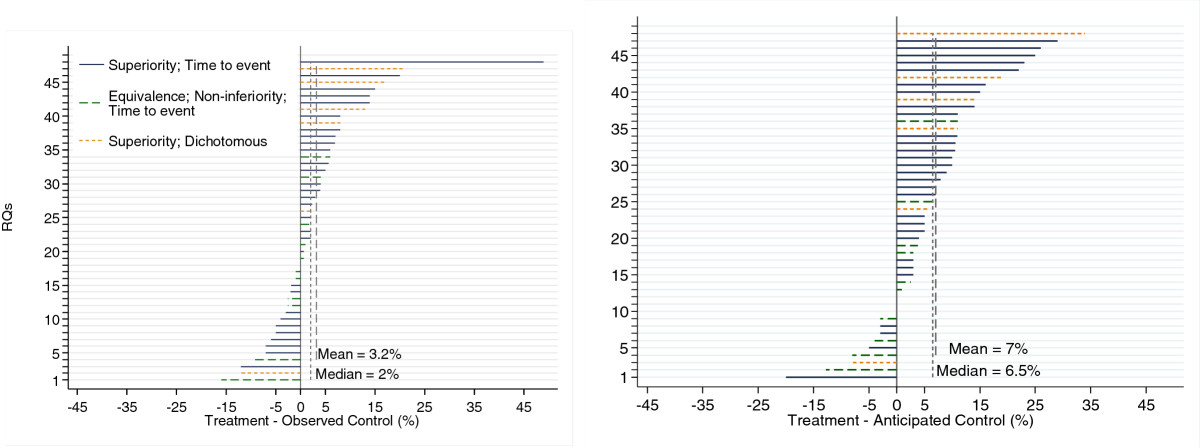
**Absolute differences for randomised question (RQ; left) and historical control (right) comparison.**

Figure 4 graphically shows the absolute differences for the randomised and HC comparisons. The overall median absolute difference (based on 48 RQs) between the experimental treatment and observed control groups was 2.0 (range: -16 to +49) in the randomised comparison and for the difference between the experimental treatment and the anticipated control group outcome was 6.5% (range: -20 to +34). The means were 3.2% (95% CI: +0.18 to +6.3) and 7.0% (95% CI: +3.9 to +10.1), respectively. A sensitivity analysis excluding seven RQs that were parts of factorial and three-arm designs did not change the results. For superiority trials with time-to-event outcomes, the median differences for randomised comparison were 2.4% (range: -12 to +49) and 7.9% (range: -20 to +29) for HC and the means were 4.1% (95% CI: +0.001 to +8.1) and 8.4% (95% CI: +4.68 to +12.1), respectively. The median difference for the equivalence or noninferiority trials was -1.0% (range: -16 to +6.0) for randomised comparison and 0.9% (range: -13 to +11) for HC; the means were -1.8% (95% CI: -6.0 to +2.4) and 0.04% (95% CI: -4.5 to +4.6), respectively. For the trials with dichotomous outcomes, the medians were 11% (range: -12 to +21) and 13% (range: -8.0 to +34) for randomised and HC, respectively; the means were 8.2% (95% CI: -4.3 to +21) and 13% (95% CI: -1.9 to +27), respectively.

### Relative differences

**Figure 5:**
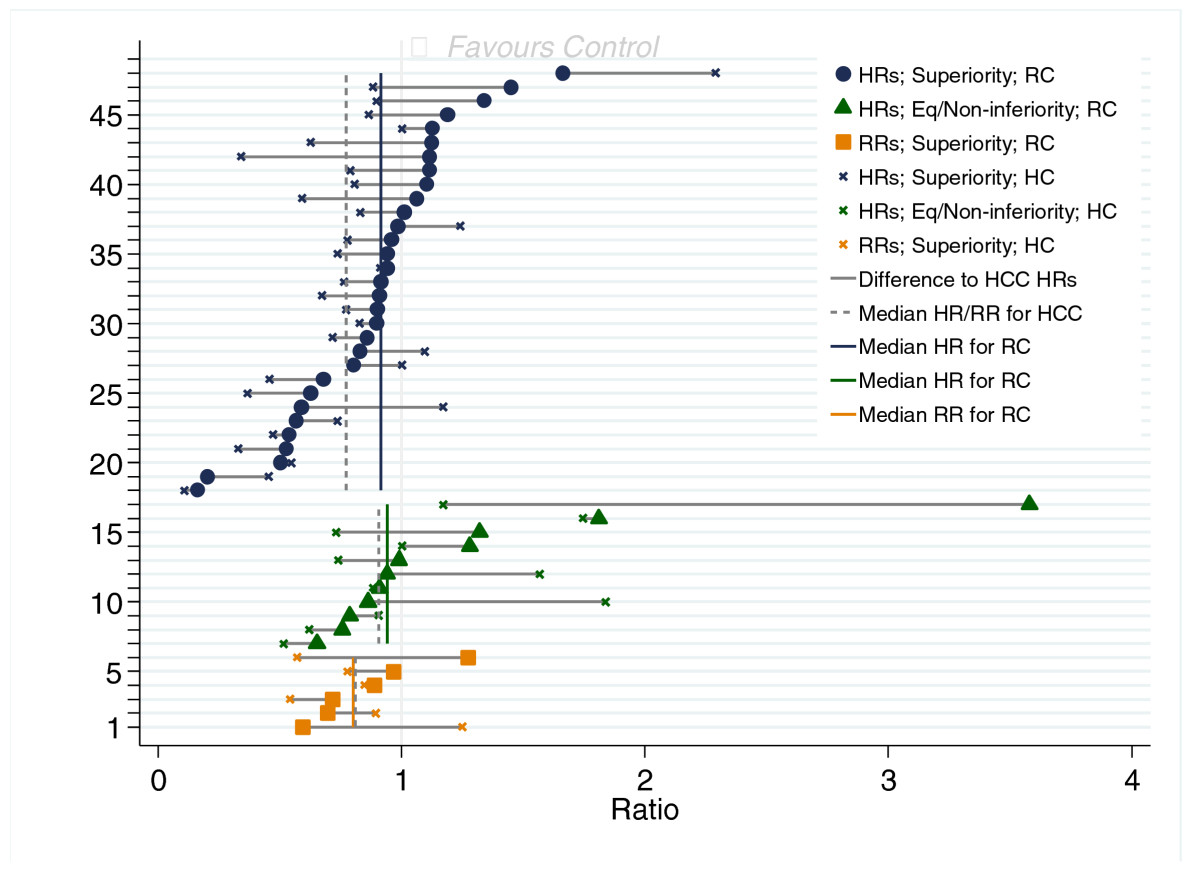
**Relative differences for historical control comparison and randomised comparison.** Solid dots, triangles or squares represent the hazard ratios (HRs) or risk ratios (RRs) for a randomised comparison. The crosses represent the HRs or RRs for the historical control comparison (HCC). The solid vertical lines show the median HRs or RRs for the randomised comparison. The dashed vertical lines show the median HRs or RRs for the HCC. For example, for the 48th question, the HR for the randomised comparison is 1.66, whereas it is 2.29 for the HCC.

Figure 5 shows HRs for time-to-event outcomes and RRs for the dichotomous outcomes for the randomised and HC comparisons. The overall median ratios for the randomised comparisons were 0.91 (range: 0.16 to 3.6) and 0.78 (range: 0.11 to 2.3) for HC; the mean values were 0.97 (95% CI: 0.83 to 1.12) and 0.85 (95% CI: 0.73 to 0.96), respectively. When we restricted this to superiority trials (*n* = 31) with time-to-event outcomes, the median HR for randomised was 0.91 (range: 0.16 to 1.7), and it was 0.77 (range: 0.11 to 2.3) for HC comparison. The means were 0.89 (95% CI: 0.77 to 1.01) and 0.78 (95% CI: 0.64 to 0.92), respectively. For equivalence/noninferiority trials with time-to-event outcomes, the median HRs were 0.94 and 0.91 for randomised and HC comparison, respectively (means: 1.26 and 1.06); for dichotomous outcomes, the RRs were 0.80 and 0.81, respectively (means: 0.86 and 0.81).

## Discussion

### Summary of findings

The potential biases in HC studies are well known and include confounding with other factors that change over time [18–20]. In this research, we attempted to quantify the effect of these biases in the field of PO. We provide evidence that the use of data from a historical population for comparison with a group of patients who received a novel treatment is likely to lead to experimental treatments’ appearing more effective than they actually are.

Of the 48 randomised comparisons analysed, we found that 73% of the control arms performed better than had been anticipated in the sample size calculation and that, on average, the control group did 3.8% better than anticipated. Furthermore, whereas the median difference was 5%, there was wide variation, with the control arm occasionally performing more than 10% better, or worse, than anticipated, meaning that, in each individual case, it would not be possible to simply adjust the anticipated control group outcome by +3.8% in HCSs. If the observed control group outcome was actually the same as that anticipated, we would expect to see random variation (due to sampling error) around this estimate (the mean difference would be zero). The fact that, based on an analysis of 48 randomised comparisons, the control group did 3.8% better on average indicates that what we are seeing is not explained by chance variation, but rather represents a systematic underestimation, on average, in the sample size calculation of the control group’s outcome.

The absolute difference between the new intervention and observed control showed that the median for the new intervention was 2.0% better than the control, whereas, when HC data were used, this difference increased to 6.5%. In relative terms, when the randomised comparison was used, the median ratio was 0.91 in favour of the treatment arm and 0.78 when the HC was used, implying that a larger treatment effect would be obtained with HCSs, potentially leading to erroneous conclusions regarding treatment efficacy.

The association between the control differences (anticipated and observed rates) and factors such as total number of patients in the trial, year of publication and disease prognosis was explored to investigate if larger differences were associated with smaller or larger trials, earlier or later trials or better or worse disease prognosis. There was a positive association between control differences and prognosis of the disease, though why this should be the case is unclear.

In HCSs, outcome data from independently conducted interventional studies are compared to derive a comparison of treatment efficacy [18, 20]. The new treatment arm, usually prospectively collected, is compared to a control group of previous patients [21] (obtained from either published or unpublished sources [22]), with the underlying assumption that the treatment effect in the HC group would represent a valid comparison for the experimental treatment. HCSs require fewer patients and are quicker and, hence, cheaper to perform than RCTs [23]. These qualities are attractive in childhood cancer research. Most childhood cancers are rare conditions, which causes difficulty because of the limited number of patients available to participate in trials, creating ethical [23, 24], statistical and funding [25, 26] challenges. However, critics of HCSs suggest that this is a much less desirable method of making comparisons and is prone to many forms of unpredictable bias, as various factors may change over time, such as supportive care, demographic characteristics and diagnostic criteria [18, 27]. This ‘temporal drift effect’ [28] results in a population-wide shift of prognostic factors questioning comparability of HC and randomised control and hence the validity of the results from HCSs.

Pinpointing the exact cause of the differences between effect estimates of observed control and anticipated control is difficult, particularly in PO, where management is often multifactorial and several factors may change over time. Changes in surgical technique (or selection of different types of patient for surgery); radiotherapy (or selection of different types of patient for radiotherapy); supportive care, such as improved antibiotic or blood product support; diagnosis (even if earlier diagnosis does not lead to better prognosis, it will result in patients appearing to live longer(time lag bias); staging (the widespread introduction of novel imaging methods means that definitions of disease stage at diagnosis and of response to treatment may change (stage migration)) may confound the historical comparison and introduce bias.

### Previous work

Differences between HCSs and RCTs have been studied previously, and the results reported reflect our present findings. In 1996, Bhansali *et al.*[29] meta-analysed HCSs and RCTs in which cisplatin-based therapies in oesophageal cancer had been assessed and found a ‘gross overestimation of treatment effect’ for cisplatin, with the historically controlled meta-analysis giving a statistically significant odds ratio (OR) of 0.32 (95% CI: 0.24 to 0.42) compared to the RCT meta-analysis OR of 0.96 (95% CI: 0.75 to 1.22). In 1986, Diehl and Perry [23] matched RCTs with HCSs covering six cancer types and compared survival rates, calculating whether there was a 10% difference in survival between the matched pairs. It was found that there was a greater than 10% difference in 38% of the pairs, and the authors concluded, ‘the assumption that historical control groups may replace randomized concurrent control groups is not valid’ (p 1114). Our results show that HCSs can overestimate treatment benefit compared to randomised comparisons by as much as 34% in absolute terms and 13% in relative terms. These larger treatment effects could be the result of selection bias, as control groups in an RCT need to meet eligibility criteria, which are generally more stringent [20]. This also indicates that the results of the HCSs are not a reflection of advantageous experimental treatment, but to a certain extent are due to a flawed design.

Some of the authors of the trials included in our data set also acknowledged the observed differences between anticipated and observed rates (for example, Souhami *et al.*[30], Evans *et al.*[31]). A good example of this is the oldest study included in our analysis, published in 1976, which underestimated the control 2-year relapse-free survival by 34%, assuming 50% and observing 84% [31]. The authors concluded, ‘[The result] points up the value of including in the study an untreated [standard treatment] control arm. An overall better survival experience obtained in the trial would have been attributed erroneously to CPM therapy [new treatment] had historical controls been used’ (p 659).

Our result differs from that of a previous study in which the researchers looked into the reporting of sample size calculation in RCTs. Charles and colleagues [32] found a median relative difference (calculated as [anticipated - observed]/anticipated) of 2.0% (interquartile range (IQR): -15% to 21%) in studies of common adult cancers, whereas we found this difference to be -7.5% (IQR: -15% to 1.5%). The HC data used in our work are probably older than the HC data used in Charles *et al.*’s set of trials; thus, the effect of the ‘temporal shift’ may be greater. This could reflect the differences in the trial types studied by Charles *et al.* and ourselves; that is, recruitment for the ones we researched, dealing with rare diseases in PO, take longer to recruit.

### Strengths and limitations

We used a reproducible methodology following a protocol, with the studies identified and the data extracted in a systematic fashion and checked by two people.

Our analyses are based on 48 RQs. However, the data represented only 50% of the published paediatric RCTs that we identified in our search. We found 92 publications describing 107 RQs with time-to-event and dichotomous outcomes. Of these, 76 RQs reported formal sample size calculations, but only 48 gave detailed parameters. This underreporting is higher than that observed by Charles and colleagues [32], who undertook a survey of how many RCTs reported sample size calculations and found that 19% of trials did not report control group assumptions adequately. One reason for this may be that Charles *et al.* conducted their survey between 2005 and 2006, whereas our studies went as far back as 1976. Indeed, the median year of publication of our included articles was 2005 versus 1995 for excluded RQs due to missing sample size information, suggesting that reporting of sample size calculations has improved in recent years.

Owing to the lack of information on the uncertainty of the HC estimate (that is, CIs around the HC estimate), in many cases, we were unable to assess whether the result of the HC scenario was consistent with that of the randomised comparison; that is, the total number of patients on which the anticipated control rate was based was not typically given. A final limitation is that for several RQs, we estimated HRs for the randomised and HC comparison using just one time point and acknowledge that these HRs would not be the same as those based on time-to-event analysis; however, this did not introduce bias, as the error could go in either direction.

### Recommendations

We and others have shown that the underlying assumption that the treatment effect in an HC group is equivalent (taking into account sampling variation) to that of a randomised concurrent control group is erroneous. Despite this, support of this study design remains popular, particularly in PO [2], with a reluctance undertake RCTs instead. This reluctance appears to stem from a rigid belief in some quarters that an RCT can be done only if there are sufficient patients for a trial to be designed using conventional parameters of 80% power and 5% α. An alternative view, to which we subscribe, is that any randomised evidence is better than none; that is, a small randomised trial will provide an unbiased estimate of the treatment effect (assuming that it is well conducted and analysed), even if it does have wider confidence intervals due to the smaller number of patients and events analysed. As we have demonstrated, the potential of HCs to introduce biases due to the confounding factors discussed above give unpredictable and often large differences in effect estimates compared to randomised concurrent controls. Hence, RCTs with alternative assumptions (for example, a relaxed α) or novel designs (for example, Bayesian) that give unbiased estimates of treatment effect should be considered whenever possible, although it needs to be accepted that, with small numbers and wide confidence intervals, cautious interpretation may be needed and definitive conclusions may not be possible. They would not be possible with HCSs either, but for additional reasons (such as biases)).

## Conclusions

Because the observed control group (that is, standard treatment arm) in RCTs did better than anticipated, we conclude that HCSs that use similar assumptions for the standard treatment are likely to overestimate the benefit of new treatments, potentially leading to children with cancer being given ineffective therapy that may have additional toxicity.

## Electronic supplementary material

Additional file 1: Extracted data.(DOC 120 KB)

Additional file 2: Scatterplot for control difference and recruited number of patients (left) and year of publication (right).(DOCX 24 KB)

Below are the links to the authors’ original submitted files for images.

Authors’ original file for figure 1

Authors’ original file for figure 2

Authors’ original file for figure 3

Authors’ original file for figure 4

Authors’ original file for figure 5

## Abbreviations

CI: Confidence interval
EFS: Event-free survival
HC: Historical control
HCS: Historically controlled study
HR: Hazard ratio
IQR: Interquartile range
OR: Odds ratio
PO: Paediatric oncology
RCT: Randomised controlled trial
RQ: Randomised question
RR: Risk ratio.
