# Supplementary material for: Comparison of anticipated and actual control group outcomes in randomised trials in paediatric oncology provides evidence that historically controlled studies are biased in favour of the novel treatment
Source: Trials. 2014 Dec 10;15:481. doi: 10.1186/1745-6215-15-481 (PMC4295234; doi:10.1186/1745-6215-15-481)
Supplement: Supplementary file 1 — Additional file 1: Extracted data.(DOC 120 KB) [file 13063_2014_2346_MOESM1_ESM.doc]

| **Additional file 1**. Extracted data | | | | | | | | | |
| --- | --- | --- | --- | --- | --- | --- | --- | --- | --- |
| **Search**  **disease term** | **First author and**  **publication year** | **Primary**  **outcome and**  **time point*** | **Design alpha** | **Design power** | **Anticipated**  **control** | **Observed**  **control** | **Observed treatment** | **Planned sample size** | **Actual sample size** |
| Wilms | Tournade 1993 [1] | DFS 2-year | 0·05 | 0·80 | 0·85 | 0·88 | 0·92 | 390 | 299 |
| Wilms | Tournade 1993 [1] | DFS 2-year | 0·05 | Not presented | 0·67 | 0·72 | 0·78 | Not presented | 123 |
| Wilms | de Kraker 2004 [2] | EFS 2-year | 0·05 | 0·80 | 0·85 | 0·914 | 0·888 | 350 | 410 |
| Neuroblastoma | Yu 2010 [3] | EFS 3-year | 0·05 | 0·80 | 0·50 | 0·46 | 0·60 | 386 | 226 |
| Neuroblastoma | London 2010 [4] | Response | 0·098 | 0·804 | 0·40 | 0·19 | 0·32 | 118 | 116 |
| Neuroblastoma | Evans 1976 [5] | RFS 2-year | 0·05 | 0·80 | 0·50 | 0·84 | 0·79 | 100 | 113 |
| Neuroblastoma | Pearson 2008 [6] | Overall Response | 0·05 | 0·75 | 0·40 | 0·53 | 0·74 | 180 | 191 |
| Rhabdomyosarcoma | Maurer 1988 [7] | Response | 0·05 | 0·90 | 0·50 | 0·623 | 0·644 | 200 | 409 |
| Rhabdomyosarcoma | Pratt 1998 [8] | Response | 0·05 | 0·80 | 0·25 | 0·56 | 0·44 | 94 | 50 |
| Rhabdomyosarcoma | Donaldson 2001 [9] | FFS 5-year | 0·05 | 0·80 | 0·65 | 0·70 | 0·70 | 438 | 490 |
| Rhabdomyosarcoma | Crist 1995 [10] | PFS 2-year | 0·05 | 0·73 | 0·80 | 0·6 | 0·77 | 92 | 95 |
| Rhabdomyosarcoma | Crist 1995 [10] | PFS 2-year | 0·05 | 0·76 | 0·70 | 0·73 | 0·70 | 314 | 171 |
| Rhabdomyosarcoma | Crist 1995 [10] | PFS 2-year | 0·05 | 0·76 | 0·70 | 0·73 | 0·75 | 314 | 176 |
| Rhabdomyosarcoma | Arndt 2009 [11] | FFS 5-year | 0·05 | 0·80 | 0·64 | 0·73 | 0·68 | 518 | 516 |
| Acute lymphoblastic leukaemia | Pieters 2007 [12] | DFS 4-year | 0·05 | 0·80 | 0·50 | 0·57 | 0·609 | 280 | 191 |
| Acute lymphoblastic leukaemia | Mitchell 2005 [13] | EFS 4-year | 0·05 | 0·99 | 0·70 | 0·78 | 0·85 | 1800 | 1603 |
| Acute lymphoblastic leukaemia | Vora 2006 [14] | EFS 4-year | Not presented | 0·99 | 0·70 | 0·85 | 0·81 | 1800 | 1492 |
| Acute lymphoblastic leukaemia | Conter 2007 [15] | DFS 4-year | 0·05 | 0·84 | 0·75 | 0·794 | 0·80 | 1700 | 2618 |
| Acute lymphoblastic leukaemia | Hill 2004 [16] | EFS 4-year | 0·05 | 0·85 | 0·65 | 0·70 | 0·72 | 800 | 1513 |
| Acute lymphoblastic leukaemia | Hill 2004 [16] | EFS 4-year | 0·05 | 0·85 | 0·45 | 0·60 | 0·54 | 240 | 374 |
| Acute myeloid leukaemia | Becton 2006 [17] | EFS 2-year | 0·05 | 0·80 | 0·35 | 0·38 | 0·42 | 560 | 565 |
| Acute myeloid leukaemia | Becton 2006 [17] | DFS 2-year | 0·05 | 0·80 | 0·45 | 0·37 | 0·45 | 560 | 418 |
| Ewings | Paulussen 2008 [18] | EFS 3-year | 0·05 | 0·85 | 0·55 | 0·47 | 0·52 | 400 | 492 |
| Ewings | Paulussen 2008 [18] | EFS 3-year | 0·05 | 0·85 | 0·70 | 0·74 | 0·73 | 200 | 155 |
| Langerhans' cell histiocytosis | Gadner 2008 [19] | Rapid Response | 0·05 | 0·76 | 0·60 | 0·63 | 0·71 | 150 | 175 |
| Osteosarcoma | Souhami 1997 [20] | OS 5-year | 0·05 | 0·80 | 0·70 | 0·552 | 0·572 | 400 | 388 |
| Osteosarcoma | Lewis 2007 [21] | OS 5-year | 0·05 | 0·90 | 0·55 | 0·55 | 0·58 | Not presented | 497 |
| Osteosarcoma | Le Deley 2007 [22] | Histologic Response | 0·05 | 0·80 | 0·5 | 0·39 | 0·56 | 206 | 234 |
| Osteosarcoma | Winkler 1988 [23] | MFS 2-year | 0·05 | 0·80 | 0·8 | 0·72 | 0·6 | 150 | 120 |
| Osteosarcoma | Goorin 2003 [24] | EFS 2-year | 0·05 | 0·80 | 0·65 | 0·75 | 0·68 | 215 | 100 |
| Osteosarcoma | Link 1986 [25] | RFS 2-year | 0·05 | 0·80 | 0·4 | 0·17 | 0·66 | 196 | 36 |
| Medulloblastoma | Taylor 2003 [26] | Survival (OS) 3-year | 0·05 | 0·90 | 0·6 | 0·761 | 0·83 | 420 | 179 |
| Medulloblastoma | Packer 2006 [27] | EFS 5-year | Not presented | 0·79 | 0·7 | 0·82 | 0·8 | 240 | 379 |
| Medulloblastoma | Bailey 1995 [28] | EFS 5-year | 0·05 | 0·80 | 0·5 | 0·598 | 0·579 | 300 | 224 |
| Medulloblastoma | Zeltzer 1999 [29] | Survival (PFS) 4-year | Not presented | 0·84 | 0·4 | 0·45 | 0·65 | 204 | 203 |
| Lymphoma | Cairo 2007 [30] | EFS 4-year | 0·20 | 0·90 | 0·88 | 0·9 | 0·8 | Not presented | 235 |
| Lymphoma | Kung 2006 [31] | EFS 3-year | 0·05 | 0·80 | 0·75 | 0·89 | 0·91 | 155 | 159 |
| Lymphoma | van-der-Werff-Ten-Bosch 2005 [32] | DFS 5-year | 0·05 | 0·80 | 0·7 | 0·8 | 0·73 | 820 | 877 |
| Lymphoma | Laver 2005 [33] | EFS 2-year | 0·05 | 0·80 | 0·75 | 0·64 | 0·7 | 273 | 175 |
| Lymphoma | Le Deley 2010 [34] | EFS 2-year | 0·05 | 0·80 | 0·62 | 0·701 | 0·725 | 204 | 217 |
| Lymphoma | Brecher 1997 [35] | EFS 2-year | 0·05 | 0·80 | 0·65 | 0·64 | 0·79 | 123 | 123 |
| Lymphoma | Woessmann 2005 [36] | FFS 1-year | 0·05 | 0·80 | 0·95 | 0·96 | 0·95 | 174 | 189 |
| Lymphoma | Woessmann 2005 [36] | FFS 1-year | 0·05 | 0·80 | 0·8 | 0·93 | 0·77 | 162 | 135 |
| Lymphoma | Link 1997 [37] | CCR2-year | 0·10 | 0·90 | 0·95 | 0·9 | 0·91 | 183 | 244 |
| Lymphoma | Lanino 2009 [38] | EFS 5-year | 0·05 | 0·80 | 0·45 | 0·5 | 0·556 | 200 | 152 |
| Lymphoma | Laskar 2004 [39] | EFS 5-year | 0·05 | 0·80 | 0·75 | 0·83 | 0·97 | Not presented | 179 |
| Lymphoma | Patte 2007 [40] | EFS long term (4-year) | Not presented | 0·90 | 0·9 | 0·934 | 0·909 | 460 | 637 |
| Lymphoma | Patte 2007 [40] | EFS long term (4-year) | Not presented | 0·90 | 0·9 | 0·919 | 0·925 | 460 | 637 |

* EFS is event-free survival; PFS is progression-free survival; RFS is relapse-free survival; second malignancy; DFS is disease-free survival; MFS is metastasis-free survival; FFS is failure-free survival; CCR is continuous complete remission; PFS is progression-free survival; OS is overall survival

**References**

1. Tournade MF, Com Nougué C, Voûte PA, Lemerle J, de Kraker J, Delemarre JF, Habrand JL, Moorman CG, Bürger D: Results of the Sixth International Society of Pediatric Oncology Wilms’ Tumor Trial and Study: a risk-adapted therapeutic approach in Wilms’ tumor. J Clin Oncol 1993, 11:1014–1023.

2. de Kraker J, Graf N, van Tinteren H, Pein F, Sandstedt B, Godzinski J, Tournade MF, the International Society of Paediatric Oncology Nephroblastoma Trial Committee: Reduction of postoperative chemotherapy in children with stage I intermediate-risk and anaplastic Wilms’ tumour (SIOP 93-01 trial): a randomised controlled trial. Lancet 2004, 364:1229–1235.

3. Yu AL, Gilman AL, Ozkaynak MF, London WB, Kreissman SG, Chen HX, Smith M, Anderson B, Villablanca JG, Matthay KK, Shimada H, Grupp SA, Seeger R, Reynolds CP, Buxton A, Reisfeld RA, Gillies SD, Cohn SL, Maris JM, Sondel PM, the Children’s Oncology Group: Anti-GD2 antibody with GM-CSF, interleukin-2, and isotretinoin for neuroblastoma. N Engl J Med 2010, 363:1324–1334.

4. London WB, Frantz CN, Campbell LA, Seeger RC, Brumback BA, Cohn SL, Matthay KK, Castleberry RP, Diller L: Phase II randomized comparison of topotecan plus cyclophosphamide versus topotecan alone in children with recurrent or refractory neuroblastoma: a Children’s Oncology Group study. J Clin Oncol 2010, 28:3808–3815.

5. Evans AE, Albo V, D’Angio GJ, Finklestein JZ, Leiken S, Santulli T, Weiner J, Hammond GD: Cyclophosphamide treatment of patients with localized and regional neuroblastoma: a randomized study. Cancer 1976, 38:655–660.

6. Pearson AD, Pinkerton CR, Lewis IJ, Imeson J, Ellershaw C, Machin D, the European Neuroblastoma Study Group, the Children's Cancer and Leukaemia Group (CCLG; formerly United Kingdom Children’s Cancer Study Group): High-dose rapid and standard induction chemotherapy for patients aged over 1 year with stage 4 neuroblastoma: a randomised trial. Lancet Oncol 2008, 9:247–256.

7. Maurer HM, Beltangady M, Gehan EA, Crist W, Hammond D, Hays DM, Heyn R, Lawrence W, Newton W, Ortega J, Ragab AH, Raney RB, Ruymann FB, Soule E, Tefft M, Webber B, Wharam M, Vietti TJ: The Intergroup Rhabdomyosarcoma Study-I. A final report. Cancer 1988, 61:209–220.

8. Pratt CB, Maurer HM, Gieser P, Salzberg A, Rao BN, Parham D, Thomas PR, Marcus RB, Cantor A, Pick T, Green D, Neff J, Jenkins JJ: Treatment of unresectable or metastatic pediatric soft tissue sarcomas with surgery, irradiation, and chemotherapy: a Pediatric Oncology Group study. Med Pediatr Oncol 1998, 30:201–209.

9. Donaldson SS, Meza J, Breneman JC, Crist WM, Laurie F, Qualman SJ, Wharam M, the Children’s Oncology Group Soft Tissue Sarcoma Committee (formerly Intergroup Rhabdomyosarcoma Group) representing the Children’s Oncology Group and the Quality Assurance Review Center: Results from the IRS-IV randomized trial of hyperfractionated radiotherapy in children with rhabdomyosarcoma—a report from the IRSG. Int J Radiat Oncol Biol Phys 2001, 51:718–728.

10. Crist W, Gehan EA, Ragab AH, Dickman PS, Donaldson SS, Fryer C, Hammond D, Hays DM, Herrmann J, Heyn R: The Third Intergroup Rhabdomyosarcoma Study. J Clin Oncol 1995, 13:610–630.

11. Arndt CA, Stoner JA, Hawkins DS, Rodeberg DA, Hayes-Jordan AA, Paidas CN, Parham DM, Teot LA, Wharam MD, Breneman JC, Donaldson SS, Anderson JR, Meyer WH: Vincristine, actinomycin, and cyclophosphamide compared with vincristine, actinomycin, and cyclophosphamide alternating with vincristine, topotecan, and cyclophosphamide for intermediate-risk rhabdomyosarcoma: Children’s Oncology Group Study D9803. J Clin Oncol 2009, 27:5182–5188.

12. Pieters R, Schrappe M, De Lorenzo P, Hann I, De Rossi G, Felice M, Hovi L, LeBlanc T, Szczepanski T, Ferster A, Janka G, Rubnitz J, Silverman L, Stary J, Campbell M, Li CK, Mann G, Suppiah R, Biondi A, Vora A, Valsecchi MG: A treatment protocol for infants younger than 1 year with acute lymphoblastic leukaemia (Interfant-99): an observational study and a multicentre randomised trial. Lancet 2007, 370:240–250.

13. Mitchell CD, Richards SM, Kinsey SE, Lilleyman J, Vora A, Eden TO, the Medical Research Council Childhood Leukaemia Working Party: Benefit of dexamethasone compared with prednisolone for childhood acute lymphoblastic leukaemia: results of the UK Medical Research Council ALL97 randomized trial. Br J Haematol 2005, 129:734–745.

14. Vora A: United Kingdom Childhood Acute Lymphoblastic Leukaemia Randomised Trial 2003 (MRC UKALL 2003). In ᅟ. Sheffield, UK: National Research Register, Research Councils UK; 2004. [http://gtr.rcuk.ac.uk/project/51DB71AF-A6F8-4A57-986D-E1A0B9EEC751] (accessed 16 November 2014).

15. Conter V, Valsecchi MG, Silvestri D, Campbell M, Dibar E, Magyarosy E, Gadner H, Stary J, Benoit Y, Zimmermann M, Reiter A, Riehm H, Masera G, Schrappe M: Pulses of vincristine and dexamethasone in addition to intensive chemotherapy for children with intermediate-risk acute lymphoblastic leukaemia: a multicentre randomised trial. Lancet 2007, 369:123–131.

16. Hill FG, Richards S, Gibson B, Hann I, Lilleyman J, Kinsey S, Mitchell C, Harrison CJ, Eden OB, the UK Medical Research Council Working Party on Childhood Leukaemia: Successful treatment without cranial radiotherapy of children receiving intensified chemotherapy for acute lymphoblastic leukaemia: results of the risk-stratified randomized central nervous system treatment trial MRC UKALL XI (ISRC TN 16757172). Br J Haematol 2004, 124:33–46.

17. Becton D, Dahl GV, Ravindranath Y, Chang MN, Behm FG, Raimondi SC, Head DR, Stine KC, Lacayo NJ, Sikic BI, Arceci RJ, Weinstein H: Randomized use of cyclosporin A (CsA) to modulate P-glycoprotein in children with AML in remission: Pediatric Oncology Group Study 9421. Blood 2006, 107:1315–1324.

18. Paulussen M, Craft AW, Lewis I, Hackshaw A, Douglas C, Dunst J, Schuck A, Winkelmann W, Köhler G, Poremba C, Zoubek A, Ladenstein R, van den Berg H, Hunold A, Cassoni A, Spooner D, Grimer R, Whelan J, McTiernan A, Jürgens H: Results of the EICESS-92 Study: two randomized trials of Ewing’s sarcoma treatment—cyclophosphamide compared with ifosfamide in standard-risk patients and assessment of benefit of etoposide added to standard treatment in high-risk patients. J Clin Oncol 2008, 26:4385–4393.

19. Gadner H, Grois N, Pötschger U, Minkov M, Aricò M, Braier J, Broadbent V, Donadieu J, Henter JI, McCarter R, Ladisch S, the Histiocyte Society: Improved outcome in multisystem Langerhans cell histiocytosis is associated with therapy intensification. Blood 2008, 111:2556–2562.

20. Souhami RL, Craft AW, Van der Eijken JW, Nooij M, Spooner D, Bramwell VHC, Wierzbicki R, Malcolm AJ, Kirkpatrick A, Uscinska BM, Van Glabbeke M, Machin D: Randomised trial of two regimens of chemotherapy in operable osteosarcoma: a study of the European Osteosarcoma Intergroup. Lancet 1997, 350:911–917.

21. Lewis IJ, Nooij MA, Whelan J, Sydes MR, Grimer R, Hogendoorn PC, Memon MA, Weeden S, Uscinska BM, van Glabbeke M, Kirkpatrick A, Hauben EI, Craft AW, Taminiau AH, the MRC BO06 and EORTC 80931 collaborators and European Osteosarcoma Intergroup: Improvement in histologic response but not survival in osteosarcoma patients treated with intensified chemotherapy: a randomized phase III trial of the European Osteosarcoma Intergroup. J Natl Cancer Inst 2007, 99:112–128.

22. Le-Deley MC, Guinebretière JM, Gentet JC, Pacquement H, Pichon F, Marec-Bérard P, Entz-Werlé N, Schmitt C, Brugières L, Vanel D, Dupoüy N, Tabone MD, Kalifa C, the Société Française d’Oncologie Pédiatrique (SFOP): SFOP OS94: a randomised trial comparing preoperative high-dose methotrexate plus doxorubicin to high-dose methotrexate plus etoposide and ifosfamide in osteosarcoma patients. Eur J Cancer 2007, 43:752–761.

23. Winkler K, Beron G, Delling G, Heise U, Kabisch H, Purfürst C, Berger J, Ritter J, Jürgens H, Gerein V: Neoadjuvant chemotherapy of osteosarcoma: results of a randomized cooperative trial (COSS-82) with salvage chemotherapy based on histological tumor response. J Clin Oncol 1988, 6:329–337.

24. Goorin AM, Schwartzentruber DJ, Devidas M, Gebhardt MC, Ayala AG, Harris MB, Helman LJ, Grier HE, Link MP: Presurgical chemotherapy compared with immediate surgery and adjuvant chemotherapy for nonmetastatic osteosarcoma: Pediatric Oncology Group Study POG-8651. J Clin Oncol 2003, 21:1574–1580.

25. Link MP, Goorin AM, Miser AW, Green AA, Pratt CB, Belasco JB, Pritchard J, Malpas JS, Baker AR, Kirkpatrick JA, Ayala AG, Shuster JJ, Abelson HT, Simone JV, Vietti TJ: The effect of adjuvant chemotherapy on relapse-free survival in patients with osteosarcoma of the extremity. N Engl J Med 1986, 314:1600–1606.

26. Taylor RE, Bailey CC, Robinson K, Weston CL, Ellison D, Ironside J, Lucraft H, Gilbertson R, Tait DM, Walker DA, Pizer BL, Imeson J, Lashford LS: Results of a randomized study of preradiation chemotherapy versus radiotherapy alone for nonmetastatic medulloblastoma: the International Society of Paediatric Oncology/United Kingdom Children’s Cancer Study Group PNET-3 Study. J Clin Oncol 2003, 21:1581–1591.

27. Packer RJ, Gajjar A, Vezina G, Rorke-Adams L, Burger PC, Robertson PL, Bayer L, LaFond D, Donahue BR, Marymont MH, Muraszko K, Langston J, Sposto R: Phase III study of craniospinal radiation therapy followed by adjuvant chemotherapy for newly diagnosed average-risk medulloblastoma. J Clin Oncol 2006, 24:4202–4208.

28. Bailey CC, Gnekow A, Wellek S, Jones M, Round C, Brown J, Phillips A, Neidhardt MK: Prospective randomised trial of chemotherapy given before radiotherapy in childhood medulloblastoma. International Society of Paediatric Oncology (SIOP) and the (German) Society of Paediatric Oncology (GPO): SIOP II. Med Pediatr Oncol 1995, 25:166–178.

29. Zeltzer PM, Boyett JM, Finlay JL, Albright AL, Rorke LB, Milstein JM, Allen JC, Stevens KR, Stanley P, Li H, Wisoff JH, Geyer JR, McGuire-Cullen P, Stehbens JA, Shurin SB, Packer RJ: Metastasis stage, adjuvant treatment, and residual tumor are prognostic factors for medulloblastoma in children: conclusions from the Children’s Cancer Group 921 randomized phase III study. J Clin Oncol 1999, 17:832–845.

30. Cairo MS, Gerrard M, Sposto R, Auperin A, Pinkerton CR, Michon J, Weston C, Perkins SL, Raphael M, McCarthy K, Patte C, FAB LMB96 International Study Committee: Results of a randomized international study of high-risk central nervous system B non-Hodgkin lymphoma and B acute lymphoblastic leukemia in children and adolescents. Blood 2007, 109:2736–2743.

31. Kung FH, Schwartz CL, Ferree CR, London WB, Ternberg JL, Behm FG, Wharam MD, Falletta JM, de Alarcon P, Chauvenet AR, the Children’s Oncology Group: POG 8625: a randomized trial comparing chemotherapy with chemoradiotherapy for children and adolescents with stages I, IIA, IIIA_1_ Hodgkin disease: a report from the Children’s Oncology Group. J Pediatr Hematol Oncol 2006, 28:362–368.

32. van der Werff Ten Bosch J, Suciu S, Thyss A, Bertrand Y, Norton L, Mazingue F, Uyttebroeck A, Lutz P, Robert A, Boutard P, Ferster A, Plouvier E, Maes P, Munzer M, Plantaz D, Dresse MF, Philippet P, Sirvent N, Waterkeyn C, Vilmer E, Philippe N, Otten J, the Children’s Leukemia Group (CLG) of the European Organisation for Research and Treatment of Cancer (EORTC): Value of intravenous 6-mercaptopurine during continuation treatment in childhood acute lymphoblastic leukemia and non-Hodgkin’s lymphoma: final results of a randomized phase III trial (58881) of the EORTC CLG. Leukemia 2005, 19:721–726.

33. Laver JH, Kraveka JM, Hutchison RE, Chang M, Kepner J, Schwenn M, Tarbell N, Desai S, Weitzman S, Weinstein HJ, Murphy SB: Advanced-stage large-cell lymphoma in children and adolescents: results of a randomized trial incorporating intermediate-dose methotrexate and high-dose cytarabine in the maintenance phase of the APO regimen: a Pediatric Oncology Group phase III trial. J Clin Oncol 2005, 23:541–547.

34. Le Deley MC, Rosolen A, Williams DM, Horibe K, Wrobel G, Attarbaschi A, Zsiros J, Uyttebroeck A, Marky IM, Lamant L, Woessmann W, Pillon M, Hobson R, Mauguen A, Reiter A, Brugières L: Vinblastine in children and adolescents with high-risk anaplastic large-cell lymphoma: results of the randomized ALCL99-vinblastine trial. J Clin Oncol 2010, 28:3987–3993.

35. Brecher ML, Schwenn MR, Coppes MJ, Bowman WP, Link MP, Berard CW, Shuster JJ, Murphy SB: Fractionated cyclophosphamide and back to back high dose methotrexate and cytosine arabinoside improves outcome in patients with stage III high grade small non-cleaved cell lymphomas (SNCCL): a randomized trial of the Pediatric Oncology Group. Med Pediatr Oncol 1997, 29:526–533.

36. Woessmann W, Seidemann K, Mann G, Zimmermann M, Burkhardt B, Oschlies I, Ludwig WD, Klingebiel T, Graf N, Gruhn B, Juergens H, Niggli F, Parwaresch R, Gadner H, Riehm H, Schrappe M, Reiter A, the BFM Group: The impact of the methotrexate administration schedule and dose in the treatment of children and adolescents with B-cell neoplasms: a report of the BFM Group Study NHL-BFM95. Blood 2005, 105:948–958.

37. Link MP, Shuster JJ, Donaldson SS, Berard CW, Murphy SB: Treatment of children and young adults with early-stage non-Hodgkin’s lymphoma. N Engl J Med 1997, 337:1259–1266.

38. Lanino E, Rondelli R, Locatelli F, Messina C, Pession A, Balduzzi A, Favre C, Santarone S, Rabusin M, Pollichieni S, Cesaro S, Dini G, Fagioli F, the AIEOP-HSCT Group: Early (day −7) versus conventional (day −1) inception of cyclosporine-A for graft-versus-host disease prophylaxis after unrelated donor hematopoietic stem cell transplantation in children: long-term results of an AIEOP prospective, randomized study. Biol Blood Marrow Transplant 2009, 15:741–748.

39. Laskar S, Gupta T, Vimal S, Muckaden MA, Saikia TK, Pai SK, Naresh KN, Dinshaw KA: Consolidation radiation after complete remission in Hodgkin’s disease following six cycles of doxorubicin, bleomycin, vinblastine, and dacarbazine chemotherapy: Is there a need? J Clin Oncol 2004, 22:62–68.

40. Patte C, Auperin A, Gerrard M, Michon J, Pinkerton R, Sposto R, Weston C, Raphael M, Perkins SL, McCarthy K, Cairo MS, the FAB/LMB96 International Study Committee: Results of the randomized international FAB/LMB96 trial for intermediate risk B-cell non-Hodgkin lymphoma in children and adolescents: it is possible to reduce treatment for the early responding patients. Blood 2007, 109:2773–2780.
