# Supplementary material for: Comparison of anticipated and actual control group outcomes in randomised trials in paediatric oncology provides evidence that historically controlled studies are biased in favour of the novel treatment
Source: Trials. 2014 Dec 10;15:481. doi: 10.1186/1745-6215-15-481 (PMC4295234; doi:10.1186/1745-6215-15-481)
Supplement: Supplementary file 2 — Additional file 2: Scatterplot for control difference and recruited number of patients (left) and year of publication (right).(DOCX 24 KB) [file 13063_2014_2346_MOESM2_ESM.docx]

**Additional file 2**. Scatter plot for control difference and recruited number of patients (left) and year of publication (right)
