# Supplementary material for: Comparison of anticipated and actual control group outcomes in randomised trials in paediatric oncology provides evidence that historically controlled studies are biased in favour of the novel treatment
Source: Trials. 2014 Dec 10;15:481. doi: 10.1186/1745-6215-15-481 (PMC4295234; doi:10.1186/1745-6215-15-481)
Supplement: Supplementary file 3 — Authors’ original file for figure 1 [file 13063_2014_2346_MOESM3_ESM.pdf]

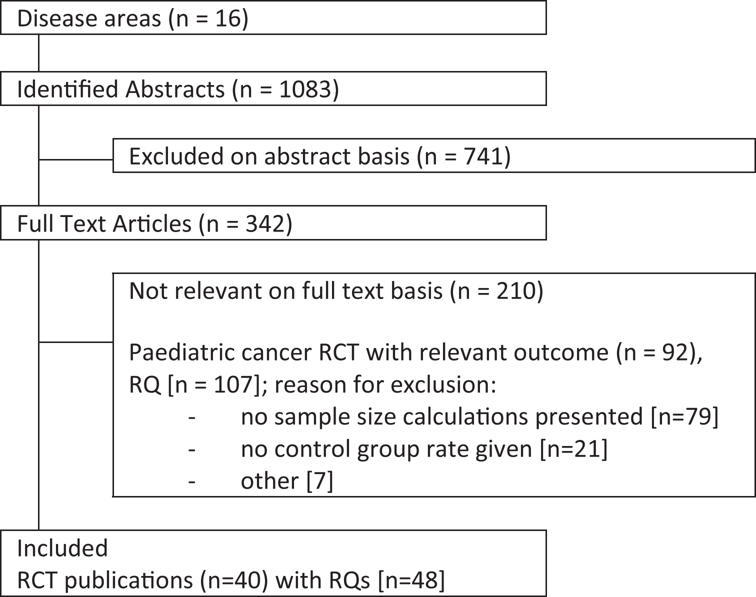
Disease areas (n = 16)
Identified Abstracts (n = 1083)
Excluded on abstract basis (n = 741)
Full Text Articles (n = 342)
Not relevant on full text basis (n = 210)
Paediatric cancer RCT with relevant outcome (n = 92),
RQ [n = 107]; reason for exclusion:
- no sample size calculations presented [n=79]
- no control group rate given [n=21]
- other [7]
Included
RCT publications (n=40) with RQs [n=48]
