# Supplementary figures and images for: Comparison of anticipated and actual control group outcomes in randomised trials in paediatric oncology provides evidence that historically controlled studies are biased in favour of the novel treatment
Source: Trials. 2014 Dec 10;15:481. doi: 10.1186/1745-6215-15-481 (PMC4295234; doi:10.1186/1745-6215-15-481)

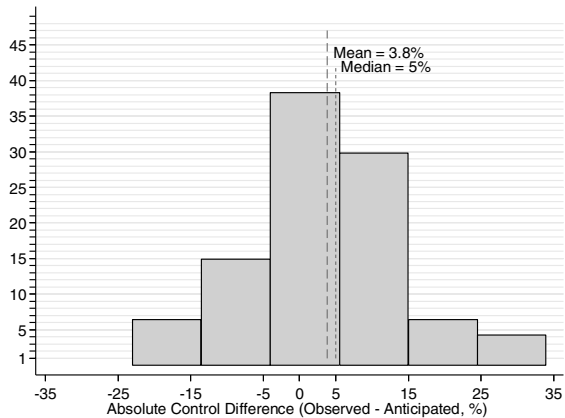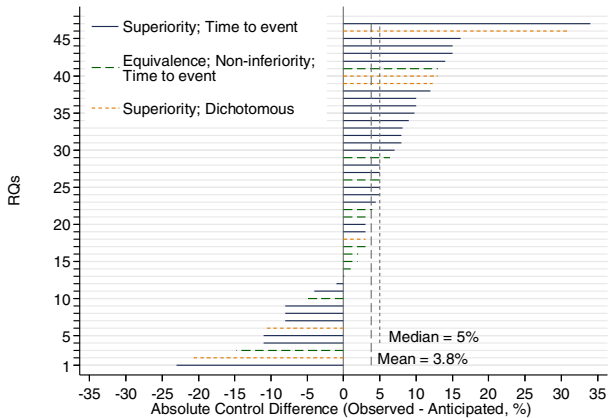

Supplement: Supplementary file 4 — Authors’ original file for figure 2 [file 13063_2014_2346_MOESM4_ESM.pdf]

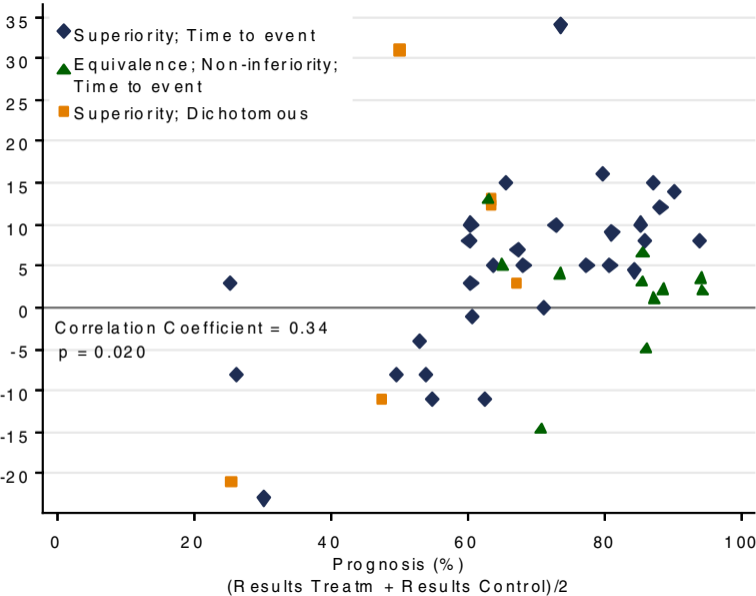

Supplement: Supplementary file 5 — Authors’ original file for figure 3 [file 13063_2014_2346_MOESM5_ESM.pdf]

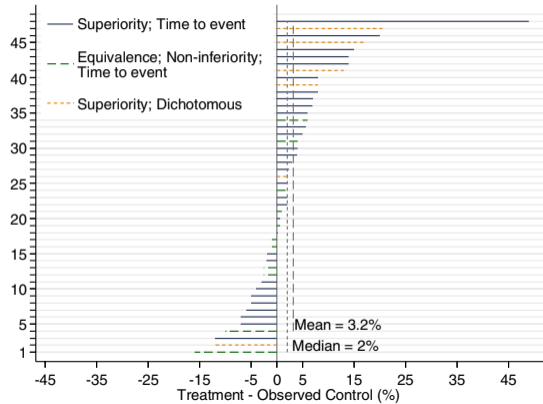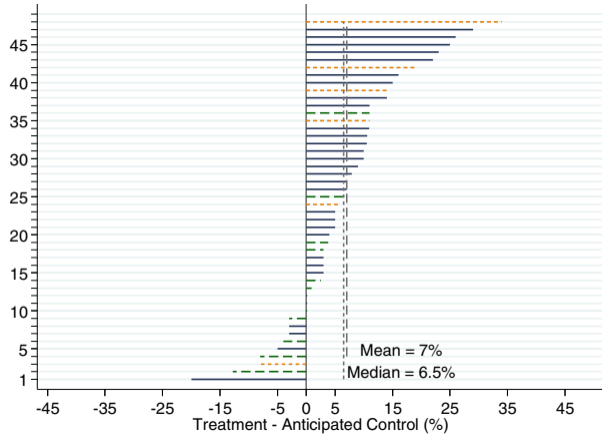

Supplement: Supplementary file 6 — Authors’ original file for figure 4 [file 13063_2014_2346_MOESM6_ESM.pdf]

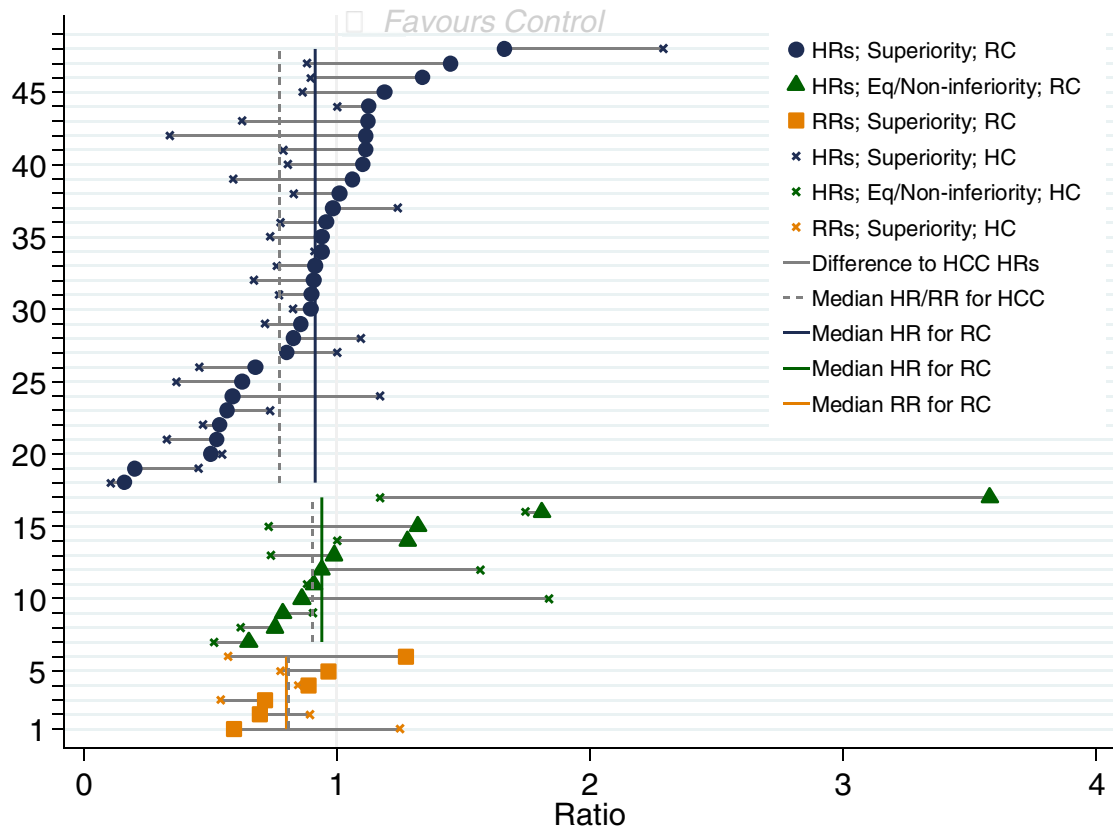

Supplement: Supplementary file 7 — Authors’ original file for figure 5 [file 13063_2014_2346_MOESM7_ESM.pdf]
